# Biological Activities Underlying the Therapeutic Effect of Quercetin on Inflammatory Bowel Disease

**DOI:** 10.1155/2022/5665778

**Published:** 2022-07-23

**Authors:** Yong-Li Lyu, Hai-Feng Zhou, Jia Yang, Fa-Xi Wang, Fei Sun, Jun-Yi Li

**Affiliations:** ^1^Nursing Department, Union Hospital, Tongji Medical College, Huazhong University of Science and Technology, Wuhan 430022, China; ^2^Department of Integrated Traditional Chinese and Western Medicine, Union Hospital, Tongji Medical College, Huazhong University of Science and Technology, Wuhan, China; ^3^The Center for Biomedical Research, Tongji Hospital, Tongji Medical College, Huazhong University of Science and Technology, Wuhan, China

## Abstract

Inflammatory bowel disease (IBD) is a chronic autoimmune disorder stemming from unrestrained immune activation and subsequent destruction of colon tissue. Genetic susceptibility, microbiota remodeling, and environmental cues are involved in IBD pathogenesis. Up to now, there are limited treatment options for IBD, so better therapies for IBD are eagerly needed. The therapeutic effects of naturally occurring compounds have been extensively investigated, among which quercetin becomes an attractive candidate owing to its unique biochemical properties. To facilitate the clinical translation of quercetin, we aimed to get a comprehensive understanding of the cellular and molecular mechanisms underlying the anti-IBD role of quercetin. We summarized that quercetin exerts the anti-IBD effect through consolidating the intestinal mucosal barrier, enhancing the diversity of colonic microbiota, restoring local immune homeostasis, and restraining the oxidative stress response. We also delineated the effect of quercetin on gut microbiome and discussed the potential side effects of quercetin administration. Besides, quercetin could serve as a prodrug, and the bioavailability of quercetin is improved through chemical modifications or the utilization of effective drug delivery systems. Altogether, these lines of evidence hint the feasibility of quercetin as a candidate compound for IBD treatment.

## 1. Introduction

Inflammatory bowel disease (IBD) is a chronic autoimmune disorder with complex etiological mechanisms and the remission-relapsing nature [1, 2]. IBD brings about physical agony to patients and imposes huge financial burden on healthcare system. Even worse, the global incidence of IBD is now rising at an accelerating rate [3, 4]. Genetic predisposition, gut dysbiosis, and environmental cues (including smoking, stress, hygiene level, UV exposure, sleep quality, and medication) are deemed to play an important part in the pathogenesis of IBD [5]. Additionally, diet-derived nutrients have been extensively investigated under the setting of IBD and are in close relationship with the immune homeostasis, epithelial integrity, and alteration of intestinal microbiota [6, 7]. Current treatments with either immunomodulators or 5-aminosalicylic acid (5-ASA) show limited efficacy, and thus, novel therapeutic approaches are eagerly needed.

Quercetin is a naturally occurring flavonol widely used as food additive [8]. The US Food and Drug Administration (FDA) approved quercetin (≥99.5%) as GRAS substance, and quercetin was allowed to be integrated into the existing food additives in Japan and Korea. Recent years, the medicinal value of quercetin gradually comes to the spotlight. Quercetin has long been recognized to possess antioxidant, antitumor, antiulcer, antidiabetic, antihypertensive, and antidepressant properties [9, 10], and the therapeutic effects of quercetin are confirmed in inflammatory disorders such as asthma, arthritis, lung injury, and diabetic angiopathy [11–13]. The regulatory roles of quercetin in IBD pathogenesis are also extensively unveiled. Dodda et al. demonstrated that quercetin exerts protective effects against the acetic acid, trinitrobenzene sulfonic acid (TNBS), and dextran sodium sulfate (DSS) induced colitis [9, 10, 14]. Quercetin counteracts the decreased body weight gain and histological destruction of colon tissue in DSS-induced colitis model [15]. Moreover, quercetin was revealed to exert therapeutic effect on *Citrobacter rodentium* infection induced colitis by enhancement of colonic microbial diversity [16]. Piling studies focus on the illumination of the beneficial outcome of quercetin in IBD treatment; nonetheless, the specific mechanisms of which have not been well documented. The present work is therefore aimed to thoroughly summarize the action modes of quercetin in IBD treatment.

## 2. The Biochemical Properties of Quercetin

Quercetin is a phytochemical compound ubiquitously present in various plants including pea, lettuce, olive, onion, apple, and medicinal herbs like sophora flower bud, cacumen biotae, galangal, and flos farfarae [17]. Unfortunately, only a trace amount of quercetin could be detected in these plants while the extraction and purification processes are difficult and costly. Before entering the human body, quercetin normally presents in the form of glycosides. In the gastrointestinal tract, the quercetin glycoside would be deglycosylated by intracellular *β*-glucosidases, which liberate the aglycon for subsequent absorption that occurs primarily by passive diffusion and secondarily through organic anion transporting polypeptide (OATP) [18]. Of note, quercetin shows relatively low water solubility and bioavailability [19]. The oral absorptive ratio of quercetin is determined by multiple factors and varies from 24% to 53% [8, 20]. The lipophilic property makes it easily penetrate the phospholipid bilayer of intestinal mucosal epithelial cell membrane [21]. Then, the intracellular quercetin is further glucuronidated, sulfated, or methylated and oxidized [22], while the bacterial ring fission of the quercetin aglycon occurs simultaneously, resulting in the breakdown of the backbone structure and the subsequent formation of smaller phenolics [23]. The half-life of quercetin is reported to be 15-28 hours [8, 24], and a research demonstrated that oral administrated quercetin does not persist in the general circulation or the enterohepatic circulation, but remain a large amount in the intestinal contents (primarily the lower bowel) [25].

## 3. Quercetin Reshapes the Commensal Microbiota in Colon

The dysbiosis of proinflammatory and anti-inflammatory colonic microbial flora and the diminished diversity of intestinal microbiome serve as the crucial detrimental factors in the pathophysiological process of IBD [26]. Quercetin was reported to enhance gut microbial diversity and rebalance the commensal microbe, thereby attenuating colitis in both *Citrobacter rodentium*-infected mice and DSS induced colitis model [15, 27]. Specifically, supplementation of quercetin facilitates the enrichment of *Bacteroides*, *Bifidobacterium*, *Lactobacillus*, and *Clostridia* and significantly reduces those of *Fusobacterium* and *Enterococcus*in [16]. Higher Shannon indices and decreased Simpson index were noticed in DSS induced colitis mice receiving dietary quercetin [27]. Mechanistically, quercetin might restore the proper intestinal host-microbe interaction by orchestrating the proinflammatory, anti-inflammatory, and bactericidal function of enteric macrophages [28]. The glycoside form of quercetin, quercitrin, was alternatively shown to suppress bacterial translocation in a rat model of experimental colitis [29], as *Escherichia coli*, *Enterococcus* spp., *Proteus* spp., and *Klebsiella pneumoniae* were identified as the downregulated gut-invasive bacteria [29]. Quercetin demonstrates huge impact on the composition of gut microbiota, and the other way around, bacteria are engaged with the production and degradation of quercetin. Expression of *β*-glucosidase in bacteria deglycosylates flavonoids into quercetin aglycon [30]. Besides, specific strains of bacteria, like *Clostridium*, *Bacteroides*, and *Eubacteria genera*, are capable of cleaving the C-ring of quercetin and releasing the 3,4-dihydroxy phenyl acetic acid and 3-(3-hydroxyphenyl) propionic acid, which also play pivotal roles in IBD pathogenesis (explained later).

## 4. Quercetin Strengthens the Intestinal Mucosal Barrier and Helps Maintain the Colonic Immune Homeostasis

The healthy colonic epithelium safeguards the homeostasis of gut immune system, facilitates the recycling of nutrients, and bolsters an integrated mucosal barrier. Host-microbial interactions participate in the remodeling of gut microenvironment [31], and so far, there are limited therapies that can efficiently restore the broken integrity of intestinal barrier [32]. Quercetin was demonstrated to temper the inflamed mucosa in IBD rats [9] and to alleviate the increased intestinal permeability induced by DSS [12]. On one hand, quercetin could elevate the expression of tight junction (TJ) proteins, boost the intestinal barrier function [14, 21], promote intestinal cell proliferation [15], and uphold the regenerative capability of intestinal mesenchymal stem cells [33]. On the other hand, quercetin reduces neutrophil and macrophage infiltration in the colon tissue of DSS-induced colitis in C57BL/6 mice [14] and inhibits production of colon damaging cytokines via suppressing the activity of myeloperoxidase (MPO), heme oxygenase-1 (Hmox1, HO-1), and inducible nitric oxide synthase (iNOS) [28, 34]. Quercetin protects enterocytes against oxidative stress-induced apoptosis, and the beneficial effect is ascribed to the elevated protein abundance of nuclear factor erythroid 2-related factor 2 (Nrf2) and increased intracellular glutathione (GSH) content [15, 35, 36]. Quercetin is also recognized as one of the few molecules that possess mast cell stabilizing function via the inhibition of calcium-dependent ATPase activity and subsequent histamine secretion [37, 38]. Additionally, quercetin could upregulate the secretory capacity of intestinal goblet cells and mucin level in gut lumen via the activation of PKC*α*/ERK1-2 signaling pathway, which provide beneficial effects on intestinal barrier integrity [39].

As described above, quercetin exhibits paramount immune regulatory function [28]. Quercetin decreases the infiltration of macrophages, neutrophils, and Th17 cells while increasing the proportion of Treg cells [14]. Also, it restrains experimental chronic intestinal inflammation by modifying the local cytokine milieu [28]. Cho et al. reported that quercetin inhibits messenger RNA expression of tumor necrosis factor alpha (TNF-*α*), IL-1*β*, and IL-6 mainly through interfering with the MAPK and NF-*κ*B pathways in lipopolysaccharide- (LPS-) stimulated macrophage [40]. Lin et al. elucidated that quercetin represses the production of proinflammatory cytokines, such as IL-17, TNF-*α*, and IL-6, and promotes the secretion of IL-10 in the colon tissues [16]. At the molecular level, miRNA is suggested to mediate the effector function of quercetin [41]. Quercetin suppresses TNF-*α* and IL-6 production by modulating the miR-369-3p/C/EBP-*β* axis in DCs, thus exerting its anti-inflammatory effect [42]. Consistently, the restraint on C/EBP-*β*; signaling of quercetin was also detected in intestinal epithelial cells, which explains its protective activity on IL-6 production elicited by heat shock response in the intestinal mucosa [43].

## 5. Quercetin Reduces the Intestinal Oxidative Stress Response

The gastrointestinal tract is a major site for generation of pro-oxidants; meanwhile, the antioxidant capacity of IBD patients is abrogated [44]. Oxidative stress is considered to exacerbate symptoms of IBD (e.g., diarrhea and abdominal pain) and contributes to the acceleration of IBD development [15]. As a potent scavenger of reactive oxygen species (ROS), the therapeutic effect of quercetin is partially ascribed to its antioxidative activity [45]. Quercetin has been demonstrated to protect Caco2 cells from hydrogen peroxide (H_2_O_2_) induced oxidative damage by elevating intracellular glutathione (GSH) content. Quercetin treatment promotes the expression of glutamate-cysteine ligase catalytic subunit (GCLC), the first rate-limiting enzyme in GSH synthesis, to eliminate excessively accumulated ROS [15]. Besides, quercetin supplementation rectifies the abnormal expression of oxidative stress markers, which are indicated by higher level of MPO, malondialdehyde (MDA), and serum nitrate (NO) concentration in colitic mice [27]. Lipid peroxides (LPO) are the secondary products of oxidative response. Treatment with quercetin results in significant decrease of LPO level and protects against the oxidative damage induced by TNBS [10]. Similarly, glycosidic form of quercetin alleviates IBD associated vascular injury via reducing the production of NO in acute TNBS induced rat colitis model [46]. The antioxidative activity of quercetin contributes to the abovementioned gut integrity strengthening effect, but considering oxidative stress is a general pathological process, these results alternatively imply that quercetin may be a suitable choice in broad spectrum of diseases (aging-related diseases, metabolic syndromes, etc.) other than IBD.

## 6. Implication of Quercetin and Its Derivatives in IBD Treatment

To enhance its translational potential for clinical IBD treatment, researchers investigated strategies that could improve bioavailability and efficiency of quercetin. Microcapsules, nanomicrovesicles, quercetin mixtures, and sugar ligands of quercetin have been extensively studied. Guazelli et al. reported that quercetin-loaded microcapsules decreases neutrophil recruitment and attenuates histological destruction in colon tissue of acetic acid-induced colitic mice, while the production of IL-10 is significantly upregulated [47]. Quercetin aglycone with monoglycosides suppresses DSS-induced colitis in mice, which is characterized by reduced oxidative stress and enhanced gut microbiota diversity [27]. Another study elucidated that quercetin loaded in silk fibroin nanoparticles reduces disease activity index (DAI) along with the expression of proinflammatory mediators (TNF-*α*, IL-1*β*, IL-6, MCP-1, ICAM-1, NLRP3, and iNOS) [48]. Moreover, heterospheroid (HS) mixture consisting of mesenchymal stem cells (MSCs) and quercetin could maximize the inflammation resolving and tissue reparative capacity as well as the engraftment efficiency of cotransplanted MSCs in colitic mice [33]. Quercetin conjugated glycol chitosan prodrug micelles or quercetin-loaded nutriose-coated vesicles are both valuable tools for the optimal treatment of IBD [49, 50]. Besides, combined quercitrin and dietary oil (rich in n-3 polyunsaturated fatty acids) exhibit synergistic effect on intestinal inflammatory remission in DSS-induced colitis model [51].

Given the free form of quercetin is present in relatively low level (a concentration of nmol/L) in human circulation [52], it is thus important to emphasize the biological activity of quercetin derivatives. The metabolic products of quercetin, generated through glucuronidation, sulfidation, methylation, and isomerization, together contribute to the overall effects of quercetin. Colonic microorganisms participate in the cycloreversion of quercetin, and 3,4-dihydroxy phenylacetic acid (3,4-DHPAA), 3-(3-hydroxyphenyl) propionic acid, and protocatechuic acid (PCA) are recognized as critical quercetin metabolites [20]. A study showed that quercetin-3-O-d-glucuronide inhibits the activity of UDP-N-acetyl glucosamine 1-carboxyvinyl transferase (MurA), which overcomes antibiotic resistance of *F. nucleatum*, a pathogenic bacterium involved in IBD progression [53]. Isorhamnetin, the methylated product of quercetin, is reported to activate human pregnane X receptor (PXR), thereby abrogating the inflammatory reaction of IBD [54]. The vicinyl dihydroxyl containing 3,4-DHPAA ameliorates oxidative stress-induced colonic damage [55], while PCA substantially inhibits cyclooxygenase-2 (COX-2) activity [56] and exerts anti-inflammatory effect on TNBS induced colitis by interfering the SphK/S1P signaling pathway [57]. Additionally, Cho et al. revealed that two quercetin derivatives, chloronaphthoquinone quercetin (CNC) and monochloropivaloyl quercetin (MCP), display supreme antioxidant properties in vitro; however, only CNC effectively decreases inflammatory damage of the colon in colitic rats [40]. Altogether, these lines of evidence support the feasibility of quercetin as a candidate chemical or prodrug for IBD treatment.

## 7. Conclusion and Perspectives

**Figure 1:**
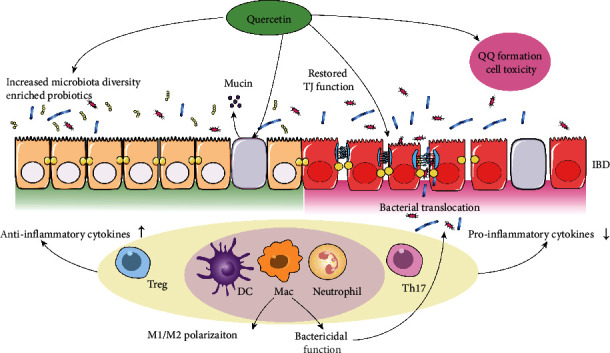
The action modes of quercetin in IBD treatment. Administration of quercetin provides beneficial anti-IBD effect through reshaping gut microbiota, stimulating the mucin production of goblet cells, strengthening the tight junction (TJ) of intestinal barrier, and restoring immune balance in colonic microenvironment. Nonetheless, administration of quercetin may bring about unwanted side effects through the generation of o-quinone/quinone methide (QQ) and subsequent cell toxicity.

**Figure 2:**
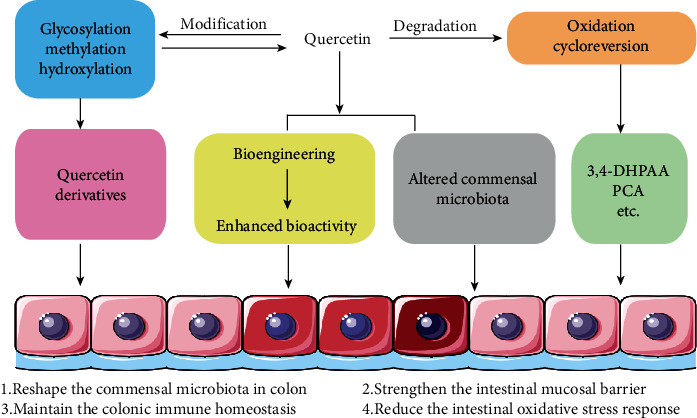
Quercetin serves as prodrug to generate bioactive derivatives. Quercetin undergoes in vivo modification and transforms into isorhamnetin and chloronaphthoquinone quercetin (CNC) that similarly exert anti-IBD effect. Intriguingly, gut microbiota is involved in the degrading process of quercetin, and the resulting products like 3,4-dihydroxy phenylacetic acid (3,4-DHPAA) and protocatechuic acid (PCA) are also potent therapeutic agents for IBD.

Quercetin is a common flavonol extracted from herbal plant, which exhibits versatile biological activities. Quercetin has proven its beneficial role in diabetes, hypertension, osteoporosis, cancer, aging etc. Under the setting of IBD, quercetin exerts the anti-IBD effect through consolidating the intestinal mucosal barrier, enhancing the diversity of colonic microbiota, restoring local immune homeostasis, and repressing the oxidative stress (Figure 1). Despite its low bioavailability, a study indicated that, an optimal plasma level of quercetin could be achieved upon repeated supplementation [58]. Alternatively, to solve the issue of low bioavailability, researchers concentrated on the modified quercetin derivatives or improving the bioavailability of quercetin via effective drug delivery system (Figure 2). Recently, the drug-intestinal flora metabolic network is regarded as a pivotal part of drug metabolism in human body. Considering the intimate interaction between quercetin and gut microbiota, there might exist individual discrepancies regarding the metabolic pathways, working mechanisms, and finally the therapeutic efficacy of quercetin after its oral administration. Thus, it is necessary to investigate the pharmacokinetics of quercetin, as the microbial flora colonized in intestinal tract varies greatly among individuals due to their different genetic background and living habits.

Of note, administration of quercetin may bring about unwanted side effects through the generation of o-quinone/quinone methide (QQ) and subsequent cell toxicity. QQ (with four tautomeric forms) is a group of oxidative products of quercetin inducing cell damage via the formation of molecular adduct. QQ is highly reactive towards thiol groups, which are abundantly present in GSH. The QQ-GSH complex termed GSQ is unstable and would release QQ in 2 min, and QQ-induced toxicity is further spread in vivo after being transported to remote areas [59]. Nonetheless, quercetin is considered safe, and numerous clinical trials have been carried out on conditions ranging from infectious diseases [60–62], tumor [63], and rheumatoid arthritis [64] to hypertension and sarcoidosis [65, 66].

Compared to other antioxidant agents (vitamin C, vitamin E, selenium, curcumin, etc.) that have proved efficacy in human IBD treatment [67], quercetin is distinguished for its alternative role as a senolytic and the metabolic regulatory function. Intermittent dosing of dasatinib plus quercetin (DQ) was tested in patients suffering from Alzheimer's disease (AD) [68], idiopathic pulmonary fibrosis (IPF) [69], and diabetic kidney disease [70], and the results were generally satisfactory. Quercetin has also demonstrated clinical values on obesity and obesity-associated metabolic syndrome [71–73]. Given obesity intertwines with IBD pathogenesis [74] and worsens the course of disease [75], those studies lay the foundation for the usage of quercetin in patients complicated with obesity-associated metabolic syndromes. However, relevant clinical studies on quercetin in human IBD are lacking and whether obese IBD patients benefit most from quercetin treatment is unclear.

To summarize, IBD is a chronic inflammatory condition where quercetin potentially provides therapeutic benefit. Nonetheless, supplementation of quercetin should be cautiously scrutinized in preclinical models before its application in human IBD patients. The long-term impact, tolerability, toxicity, and efficacy (dosage, timing, and expenditure) of quercetin all demand further investigations.

## Data Availability

All data needed to evaluate the conclusions in this article are included in the paper and/or its supplementary information. Additional data related to this paper may be requested from the authors.
